# Efficacy and Safety of Camrelizumab in Combination with Docetaxel + S-1 Sequenced by Camrelizumab + S-1 for Stage III (PD-1+/MSI-H/EBV+/dMMR) Gastric Cancer: Study Protocol for a Single-Center, Prospective, Open-Label, Single-Arm Trial

**DOI:** 10.3389/fsurg.2022.917352

**Published:** 2022-06-28

**Authors:** Zhongyi Dong, Bo Ni, Linxi Yang, Yujing Guan, Chunchao Zhu, Enhao Zhao, Gang Zhao, Xiang Xia, Zizhen Zhang

**Affiliations:** Department of Gastrointestinal Surgery, Renji Hospital, School of Medicine, Shanghai Jiao Tong University, Shanghai, China

**Keywords:** camrelizumab, docetaxel, gastric cancer, S-1, clinical protocols

## Abstract

**Background:**

Gastric cancer occupies the fourth highest morbidity rate of cancers worldwide. A higher incidence of gastric cancer had been found in East Asia compared to the other regions. Gastrectomy with radical lymph node dissection is the cornerstone of curative treatment for Stage III gastric cancer, and postoperative systemic chemotherapy with docetaxel, S-1 improved patients’ disease-free survival rates. However, advances in immunotherapy bring innovations in the management of patients with gastric cancer. The objective of this study was to explore the efficacy and safety of camrelizumab in combination with docetaxel + S-1, sequenced by camrelizumab + S-1 in stage III gastric cancer patients who are EBV positive, with defective mismatch repair and CPS ≥5.

**Methods and analysis:**

This prospective, open-label, single-arm trial was performed at Renji Hospital. In this study, a total of 70 adult patients aged 18–80 years with Stage III (PD-1+/MSI-H/EBV+/dMMR) gastric cancer confirmed by post-operative pathology will be enrolled after screening. Participants will receive the specific chemotherapy regimen until 1 year after the operation or until tumor recurrence or metastasis. The primary outcome is the 3-year disease-free survival rate measured by the Clopper-Pearson method and 95% confidence intervals. The secondary outcomes include overall survival, incidence and severity of adverse effects, and laboratory abnormalities. The data will be analyzed by the Kaplan-Meier method and log-rank test. The patients will be followed up every 3 months with imaging investigation until clinical remission.

**Ethics and dissemination:**

All participants will provide informed consent. The protocol has been approved by the Shanghai Jiaotong University School of Medicine, Renji Hospital Ethics Committee (KY2019-191). The results will be disseminated through peer-reviewed manuscripts, reports and presentations.

**Clinical Trial Registration:**

ClinicalTrials.gov, identifier: ChiCTR1900027123. Registration date November 2019; first enrolment December 2019; expected end date December 2021; trial status: Ongoing.

**Brief Abstract:**

A clinical trial for Stage III (PD-1+/MSI-H/EBV+/dMMR) gastric cancer patients who accepted anti-PD-1 therapy combined with docetaxel + S-1 as the first-line treatment and explored improvements in three-year disease-free survival rate.

## Background

Gastric cancer is one of the most common malignant tumors, accounting for 10%–15% of systemic malignancies (1). Its morbidity and mortality are ranked 4^th^ and 2^nd^ respectively in the world and second highest in China (2). Radical gastrectomy, D2 lymph node dissection and systemic chemotherapy were performed for advanced gastric cancer, whereas D1 + lymph node dissection with partial omentectomy, were performed for early gastric cancer (3). In addition to these standardized therapies, molecular-targeted drugs such as the human epidermal growth factor 2 (Herceptin) and the anti-angiogenic drug lapatinib have been gradually recognized in recent years. However, due to the high heterogeneity of gastric cancer and the lack of available targets, the clinical application of these drugs is still limited (4). With the development of research on Programmed Death Receptor-1(PD-1) and its ligand (PD-L1), immunotherapy has gained the attention of researchers in cancer treatment.

Multiple clinical studies have been carried out in the treatment of gastric cancer with immune checkpoint blockade therapy. Pembrolizumab, a PD-1 inhibitor binds PD-1, blocking the activation of PD-1 and PD-L1/2 signaling, and has been approved for melanoma treatment (5). A series of studies, such as KEYNOTE-012 and KEYNOTE-059, have confirmed its efficacy in advanced esophageal and gastric cancer with PD-L1-positive expression (6, 7). In KEYNOTE-062, all patients had a combined positive score (CPS) ≥1, and neither Pembro alone nor Pembro-Chemo significantly improved survival. However, for patients with CPS ≥ 10, the 12-month OS rate was 80% compared to 57% in this study (8). Based on these findings, PD-L1-positive patients are more likely to benefit from PD-1 immunotherapy, and the FDA has granted the accelerated approval of pembrolizumab for advanced gastric cancer treatment (7). In the treatment of metastatic colorectal cancer, the researchers found that patients with defective mismatch repair (dMMR) benefited more from PD-1 treatment than patients with proficient mismatch repair (pMMR), with some achieving lasting remission. A clinical trial conducted by Le et al. (9) not only demonstrated that gastric cancer patients with dMMR benefited more from PD-1 therapy but also confirmed that dMMR is an effective biomarker for response to PD-1 treatment. Panda et al. (10) reported PD-L1 antibody therapy that Epstein-Barr virus (EBV)-positive gastric cancer patients were treated with PD-L1 antibodies after the failure of standard multiline therapy. The analysis of tumor tissue specimens showed that it was negative for microsatellite instability and positive for EBV, suggesting that EBV might be a biomarker indicating sensitivity to PD-1 therapy.

In summary, this study will include patients with stage III gastric cancer who are EBV positive and have defective mismatch repair, CPS ≥5. These patients’ response to traditional chemotherapy is not usually satisfactory and limited current means to effectively treat such patients (11, 12). We believe that our regimens may bring unexpected relief to them. And the study is designed to investigate the effects of immunotherapy combined with adjuvant chemotherapy on the 3-year disease-free survival (DFS) rate and overall survival (OS) in patients with stage III gastric cancer.

## Methods and Analysis

### Study Design

The study was designed as a single-center, single group, prospective, open-label and single-arm research at Renji Hospital, Shanghai Jiao Tong University between Jan. 2020 and Dec. 2021. The data collection and analysis were completed to evaluate the efficacy and safety of camrelizumab in combination with docetaxel + S-1, sequenced by camrelizumab + S-1 as adjuvant therapy in stage III gastric cancer (PD-L1 + / MSI-H / EBV +/dMMR). The trial was in compliance with the applicable regulations and the Helsinki Declaration. Furthermore, the protocol has been approved by the Shanghai Jiaotong University School of Medicine, Renji Hospital Ethics Committee (KY2019-191) and registered in the Chinese Clinical Trial Registry (ChiCTR1900027123).

### Study Participants

**Figure 1:**
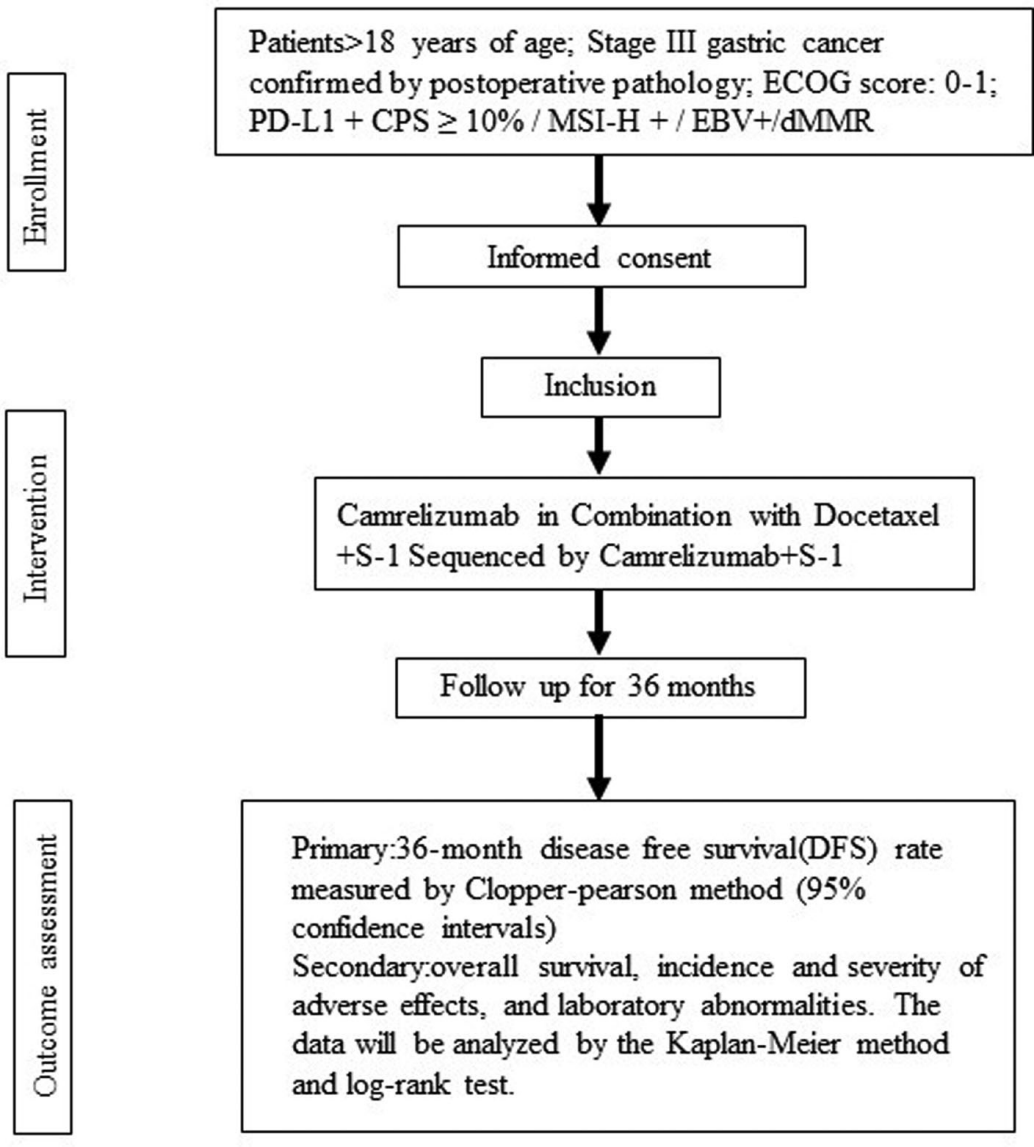
Flowchart summarizing the trial procedure. Stage III gastric cancer participants in Camrelizumab in Combination with Docetaxel + S-1 Sequenced by Camrelizumab + S-1 for trial.

The written informed consent will be provided before starting the trial and then the participants will be screened by a gastrointestinal surgeon to establish whether they meet the eligibility criteria. The qualified patients will be admitted within 4 weeks after surgery and begin treatment. During the follow-up period, the patients will be scheduled for a return visit every 3 weeks and an imaging evaluation every 4 weeks until the end of the treatment. They will be assessed for the primary and secondary outcomes after the intervention and during the 3-year follow-up. The end of the trial is defined as the last follow-up date for the last patient. Figure 1 summarizes the flow of participants.

### Inclusion and Exclusion Criteria

Complete inclusion/exclusion criteria are detailed in Table 1.

**Table 1:** Inclusion and exclusion criteria.

Inclusion Criteria	(1) The patients voluntarily joined the study and signed the informed consent
	(2) ≥ 18 years old, ≤80 years old
	(3) Stage III gastric cancer confirmed by pathology
	(4) ECOG score: 0–1
	(5) Detection of biomarkers in postoperative gastric cancer samples demonstrating PD-L1 + CPS ≥ 10% / MSI-H + / EBV+/dMMR
	(6) During the study treatment period and within 3 months after the end of the study treatment period, a medically recognized contraceptive measure (such as IUD, contraceptive pill or condom) should be used by the patients
	(7) The baseline blood routine and biochemical indexes of the selected patients should meet the specified standard
Exclusion Criteria	(1) Pregnant or lactating women; women of childbearing age with a positive pregnancy test
	(2) Have other malignant tumors in the past 5 years. Distant metastasis was diagnosed by CT /MR/ EUS
	(3) Received previous anti-tumor treatment
	(4) Uncontrollable pleural effusion, pericardial effusion, ascites or severe cardiovascular diseases
	(5) With gastroduodenal obstruction/bleeding, digestive dysfunction or malabsorption syndrome
	(6) Complicated with severe uncontrolled concurrent infection or other serious uncontrolled concomitant diseases, moderate or severe renal injury
	(7) Allergic reaction to the drugs used in this study
	(8) Steroid or other systemic immunosuppressive therapy was used within 14 days before admission
	(9) Patients who received study drug treatment within 4 weeks before enrollment
	(10) Active autoimmune diseases
	(11) History of primary immunodeficiency
	(12) Immunosuppressive drugs were used within 4 weeks prior to the first dose of study treatment
	(13) Received live attenuated vaccine within 4 weeks before the first dose of study treatment or during the study period
	(14) Active tuberculosis
	(15) History of allogeneic organ transplantation and allogeneic hematopoietic stem cell transplantation
	(16) HIV antibody positive, active hepatitis B or C
	(17) Other factors that may affect the safety or test compliance of the subjects according to the judgment of the researchers

### Intervention

#### Camrelizumab

Camrelizumab (200 mg) was administered intravenously for 30 min (not less than 20 min, not more than 60 min, including flush time) every 3 weeks. Repeated every 21 days and 3 weeks for a course. The drug was discontinued 1 year after the operation or with tumor recurrence or metastasis, determined by imaging.

Camrelizumab dosing adjustment will not be allowed. However, if the patient develops severe complications, the researchers can suspend the medication.

#### Tegafur Gimeracil Oteracil Potassium

Tegafur 80–120 mg (adjusted dose by the researcher according to the patient’s tolerance) d1-d14 taken orally followed by resting for 7 days. The cycle will be repeated every 21 days. And the course of treatment is 3 weeks and the drug will be discontinued 1 year after the operation or on tumor recurrence or metastasis, determined by imaging.

According to the patient’s condition, the researcher will decide to adjust the drug dosage. Each dose was increased or decreased in the order of 40 mg, 50 mg, 60 mg and 75 mg. If the 40 mg dose is not tolerated, the drug administration will be discontinued.

#### Docetaxel

Docetaxel 40 mg/m2 (the dose is adjusted by the researcher according to the patient’s tolerance level) is given intravenously for 1 h every 3 weeks from the second cycle to the seventh cycle. The drug was discontinued 1 year after the operation or on tumor recurrence or metastasis, determined by imaging.

The white blood cell count should be monitored regularly during the treatment. Docetaxel will only be given to patients with a neutrophil count greater than 1500/mm^3^. If severe neutropenia occurs (<500/mm^3^ for 7 days or more), a reduction in dosage is recommended in the next course of treatment. If there is no improvement, further dose reduction or discontinuation will be recommended.

The treatment protocol described above will be given continuously for 1 year after the operation or until tumor recurrence or metastasis, determined by imaging. Safety visits and follow-ups will be performed after treatment. Cancer progression will also be followed up after the end of treatment for subjects who have non-disease progression and have completed the treatment.

### Outcome Measures

#### Primary outcome

To observe and evaluate the 3-year disease-free survival (DFS) rate in patients with stage III gastric cancer (PD-L1 + / MSI-H / EBV +/dMMR).

#### Secondary outcome

1. To observe and evaluate the overall survival (OS) in patients with stage III gastric cancer (PD-L1 + / MSI-H / EBV +/dMMR).
2. To observe and evaluate the safety and adverse events in patients with stage III gastric cancer (PD-L1 + / MSI-H / EBV +/dMMR).

### Sample Size Calculation

The sample size calculation will be based on the 3-year disease-free survival (DFS) rate of the included patients. The study was designed as a prospective single-arm trial, focusing on patients with stage III gastric cancer (PD-L1+/MSI-H/EBV+/dMMR). The sample size was estimated based on the following assumptions. According to previous literature, standard treatment has a 3-year disease-free survival rate of 67% (13). We estimate that PD-1 combined with adjuvant chemotherapy could result in a rate of 81%. It is expected that the studied regimen will achieve remission for a greater proportion of patients. Based on a two-tailed test of two independent means with a significance (α) level of 0.05% and at least 80% of the test efficacy, the number of participants determined was 62. If the loss rate does not exceed 10%, the minimum sample size for this study is 70 patients.

### Statistical Analysis

The statistical analysis will be based on intention-to-treat (ITT) data or safety analysis set (SAT). The demographic characteristics of each group will be compared using the independent t-test and the chi-squared test for continuous variables and dichotomous variables. Measurement data will be expressed as mean, standard deviation, median, maximum, minimum. Enumeration data and ranked data will be expressed as a constituent ratio, rate, confidence interval. All statistical data will be analyzed by SPSS. The disease-free survival (DFS) rate will be estimated by Clopper-Pearson and the 95% confidence interval will be calculated. The overall survival (OS) rate will be analyzed by the Kaplan-Meier method and log-rank test. TTR is described by mean, standard deviation, median, maximum and minimum. Tumor markers associated with anti-PD-1 antibodies, such as levels of PD-L1 or other biomarkers in tumor tissue, will also be analyzed using descriptive statistics.

### Data Collection and Monitoring

Data collection will include patient demographics and disease subclassification based on the Montreal Classification, such as clinical phenotype, disease location and surgical history. The collected patient demographic data will include the identity card number, sex, date of birth, age at diagnosis, duration, nationality, height and weight. Laboratory tests (including blood, urine, fecal occult blood test, liver/kidney function, blood glucose, serum electrolytes, serum proteins, clotting tests, thyroid function, tumor markers, etc.) should be completed within 14 days prior to the first treatment cycle Tumor imaging (including chest, abdomen, and pelvis CT scans; head CT or MRI and bone scans if clinically necessary) and echocardiography will be completed within 28 days. The participants and specific follow-up staff will screen for infectious diseases such as HIV, hepatitis B virus and hepatitis C virus.

Follow-up will be conducted by specific staff for all the participants enrolled in this study. The follow-up program will be established after the surgery and will be performed annually thereafter. In principle, all the examinations were suggested to be carried out in Renji Hospital. If the patients are reexamined in other hospitals, grade III, first-class hospitals are recommended and the follow-up staff will track and record the results. The tests included: (1) Adverse reactions; (2) Physical examination: superficial lymph nodes, abdomen, metastatic signs and so on; (3) Peripheral blood routine examination: HB, RBC, WBC, LYM, NEU, NEU%, PLT; (4) Blood biochemistry: albumin, prealbumin, total bilirubin, AST, ALT, creatinine, urea nitrogen, fasting blood-glucose; (5) Serum tumor markers: CEA, CA19-9, CA72-4; (6) Abdominal and pelvic CT.

All participants should be evaluated for safety and adverse events within 90 days of the last administration, and the patients’ condition should be recorded. The following examinations should be performed on the 90th day of follow-up after treatment. (1) ECOG score standard; (2) Blood routine examination: hemoglobin, red blood cell, white blood cell, neutrophil count, and so on; (3) Routine urine test; (4) Blood biochemistry; (5) Evaluation for adverse events.

Combined with these results, the researchers assessed and recorded the postoperative survival status of all patients to determine tumor recurrence or metastasis. If patients refuse to be followed up according to our protocol, they will be recorded as missing cases and analyzed with those patients who met the study criteria at the end of the study.

After completion of the 90-day follow-up after discontinuation, the patients will enter the survival follow-up period. The participants, their family members, or local physicians are interviewed by telephone at least every three months. Follow-up staff will collect information on survival (date of death and cause of death) and post-treatment until either the end of death or the subject were lost to follow-up or the study was terminated by the researchers (Table 2).

**Table 2:** Check and visit schedule.

Check/Visit	Screening^a^	1st cycle (21d)	2nd cycle (21d)	3rd cycle (21d)	4th cycle (21d)	5th cycle (21d)	6th–18th Cycle (21d)	Follow-up period		
		First Week C1/D1	Forth Week C2/D1(±3d)	Seventh Week C3/D1(±3d)	Tenth Week C4/D1(±3d)	Thirteenth Week C5/D1(±3d)	N week C6-C18/D1	Finish/ Quit	Safety (every 30ds)	Survival (every3 mons)
ECOG Score	**√**	**√**	**√**	**√**	**√**	**√**	**√**	**√**	**√**	** **
Physical Examination	**√**	** **	** **	** **	** **	**√**	**√**	**√**	**√**	** **
Vital signs	**√**	**√**	**√**	**√**	**√**	**√**	**√**	**√**	**√**	** **
Blood routine^b^	**√**	** **	**√**	**√**	**√**	**√**	**√**	**√**	**√**	** **
Urine Routine^c^	**√**	** **	**√**	**√**	**√**	**√**	**√**	**√**	**√**	** **
Blood Biochemical^d^	**√**	** **	**√**	**√**	**√**	**√**	**√**	**√**	**√**	** **
Occult blood test^e^	**√**	** **	** **	** **	** **	** **	** **	** **	** **	** **
Coagulation test^f^	**√**	** **	** **	** **	** **	**√**	**√**	**√**	** **	** **
Thyroid function test^g^	**√**	** **	** **	** **	** **	**√**	**√**	**√**	** **	** **
Pituitary-adrenal test^h^	**√**	** **	** **	** **	** **	** **	** **	** **	** **	** **
ECG^i^	**√**	** **	**√**	**√**	**√**	**√**	**√**	**√**	** **	** **
Imaging Evaluation^j^	**√**	** **	** **	** **	** **	**√**	**√**	**√**	** **	** **
Pregnancy test^k^	**√**	** **	** **	** **	** **	** **	** **	**√**	** **	** **
Adverse Events^l^	**√**	**√**	**√**	**√**	**√**	**√**	**√**	**√**	**√**	**√**
Tumor Markers^m^	**√**	** **	** **	** **	** **	**√**	**√**	** **	** **	** **
Quality of Life Score	**√**	**√**	** **	**√**	** **	**√**	**√**	**√**	** **	** **
Compliance	**√**	** **	** **	** **	** **	** **	** **	**√**	** **	** **

^a^ *Screening: Tests and evaluations should be completed within 14 days prior to the first use of the drug. Tumor imaging and echocardiography can be completed in 28 days.*

^b^ *Blood routine should include complete blood count and classification. If necessary, the researchers can perform additional tests.*

^c^ *Routine urine tests include white blood cells, red blood cells, and urine proteins. Two consecutive urine protein test 2 + ∼ 3 +, or proteinuria 4 +, requires 24-hour urinary protein quantification. Subjects with 24-hour urinary protein quantification more than 1 g during the screening period are not recommended.*

^d^ *Blood biochemistry: Including ALT, AST, GGT, AKP, LDH, TP, ALB, TBIL, DBIL, GlU, IBIL, BUN or urea, Cr, K ^+^, Na ^+^, Cl^-^, Ca^2+^, Mg^2+^ . If necessary, the researchers can perform additional tests.*

^e^ *Occult blood test: Eliminate dietary interference.*

^f^ *Coagulation test: INR, APTT, PT, FIB.*

^g^ *Thyroid function test: Serum FT3, FT4, TSH.*

^h^ *Pituitary-adrenal test: Including Corticotropin, cortisol, sex hormones test.*

^i^ *12-lead ECG: This should be completed before each administration of Camrelizumab. (Noting Qt, QTc, and P-R intervals)*

^j^ *Imaging Evaluation: Baseline and postoperative radiographic evaluation were performed according to RECIST V1.1.*

^k^ *Pregnancy test: Women of child-bearing age should perform blood or urine tests during the screening period (72 hours before first administration) and at the time of completion or withdrawal from the study.*

^l^ *Adverse events: All adverse events were observed and recorded according to NCI CTCAE V5.0 standards.*

^m^ *Tumor markers: CEA, CA199 and CA72-4 were detected before the first administration, every 4 cycles and the end of treatment.*

### Safety and Adverse Event Monitoring

An adverse event (AE) is any undesirable clinical event that occurs to a study participant during the study but does not necessarily have a causal relationship with the treatment. AEs may include any unexpected adverse symptom, sign, laboratory abnormality, or disease. For example:

(1) The original medical condition/disease is aggravated prior to entry into the clinical trial;
(2) Any new adverse medical condition (including symptoms, signs, recently diagnosed disease);
(3) Abnormal laboratory results with clinical significance.

The researchers will keep detailed records of any AE in the subjects.

Severe adverse event (SAE) refers to a medical event occurring during a clinical trial that requires hospitalization or prolonging the current hospitalization, causes persistent or significant disability, affects working capacity, endangers life, or results in congenital malformation/birth defect and other medical events. Hospitalization events exclude rehabilitation facilities, nursing homes, admission to a routine emergency room, day surgery (outpatient/ambulatory) and social reasons. Furthermore, if hospitalization or prolonged stay is unrelated to the exacerbation of AE, these events should not be included under SAE, such as annual physical examination, admission for pre-existing disease and so on. First, the subjects should sign an informed consent effective until 90 days after the last drug administration. The researchers should keep detailed records of symptoms, severity, association with the experimental drug, time of occurrence and treatment, measures that have been taken, time and manner of follow-up, and outcome. Any severe adverse event should immediately be reported by completing the SAE Report Form provided by the CFDA. Then the researcher should report the condition to the provincial, autonomous regional and municipal medical products administration, CFDA, and health administrative departments within 24 h and timely report to the ethics committee. SAEs occurring after 90 days following the last drug administration are generally not reported unless suspected to be related to the trial.

All AE/SAE should be followed up until disappearance, remission to baseline level, reaching a stable state, or a reasonable explanation (such as loss to follow-up, death).

### Ethics and Dissemination

The study has been approved by Shanghai Jiaotong University School of Medicine, Renji Hospital Ethics Committee (KY2019-191) and registered in the Chinese Clinical Trial Registry (ChiCTR1900027123). The trial will be conducted according to the principles of the Declaration of Helsinki and in accordance with Good Clinical Practice (GCP) standards. Informed consent will be obtained by the coordinators or researchers associated with this protocol. It details the drug use, the course of the study, and the risks of the study are fully explained. Written informed consent must be obtained before proceeding with the study. Throughout the course of the clinical study, participants could withdraw their consent at any time. All the personal information about the subjects will be kept strictly confidential.

Results will be published in the open-access peer-reviewed medical literature as well as submitted for presentation at national and international meetings.

## Discussion

For decades, chemotherapy has remained the mainstay of effective treatment for advanced GC (14). For more than 50 years, the vast majority of chemotherapy regimens for gastric cancer have been based on the infusion of 5-FU (15). Although molecular targeted therapy is rapidly evolving in individualized and precision medicine, only a few drugs have successfully been considered over conventional treatment (16). The CSCO guidelines suggest that patients with resectable gastric cancer receive postoperative adjuvant treatment. Furthermore, XELOX or S-1 alone are recommended for grade I tumors (17). In addition, S-1 is an oral 5-FU prodrug and has been widely accepted for the treatment of advanced GC in Japan (15). The JCOG9912 randomized trial showed that oral S-1 alone was as effective as a continuous infusion of fluorouracil for patients with metastatic gastric cancer (18). The main component of S-1 is tegafur with two enzyme inhibitors:5-chloro-2,4-dihydroxypyridine and potassium oxonate (19). It has been reported that CDHP enhances the anti-cancer activity of tegafur and Oxo blocks 5-FU phosphorylation, reducing local toxicity (20). Although S-1 monotherapy reduced the incidence of peritoneal metastasis, approximately 20%–30% of patients relapsed after one year (21).

Docetaxel, a cytotoxic anti-cancer agent, is a notable treatment option available for patients with advanced gastric cancer (22). Docetaxel can impair mitosis and induce apoptosis by suppressing the microtubule dynamics of the mitotic apparatus (23). It has not only revealed promising activity as a single agent but also showed efficacy in combination with S-1 (24). Several clinical trials of docetaxel in combination with S-1 have been conducted to explore the superiority over S-1 monotherapy. The randomized phase III study “JACCRO GC-07” (13), which compared S1/docetaxel vs. S-1 alone in 915 enrolled patients, showed significant superiority of S-1 plus docetaxel (65.9%) to S-1 (49.6%) for 3-year relapse-free survival (hazard ratio, 0.632; 99.99% CI, 0.400 to 0.998; stratified log-rank test, *P* < .001).

Although the DS regimen assessment results reported significant benefits for the patients, the advances in immunotherapy bring considerable changes. The “FORCE1” study (25) suggests that a large percentage of Chilean patients may be candidates for immunotherapy because of the prevalence of Epstein-Barr virus–related gastric cancer leading to microsatellite instability. Although only 5%–10% of adenocarcinomas are Epstein-Barr-virus (EBV)-associated gastric cancer worldwide, it is commonly believed that these patients have better disease courses and overall outcomes than Epstein-Barr negative patients. They potentially benefit from immunotherapy and have higher chances of survival (7).

Based on the results of these clinical studies, although guidelines recommend camrelizumab as a third-line treatment for gastric cancer, we would like to explore its efficacy and safety as a first-line treatment for patients with specific phenotypes of gastric cancer. To our knowledge, these strategies have not been compared in a clinical trial to date. Therefore, this study aims to investigate if camrelizumab, in combination with docetaxel + S-1, sequenced by camrelizumab + S-1treatment, can improve the outcome of patients who are EBV positive with defective mismatch repair and CPS ≥5.

However, this research has some shortcomings. Firstly, the number of eligible patients is relatively small. It may take long time and much money to enroll all patients. Secondly, the study we conducted is exploratory and subsequent large-scale clinical studies are still needed to provide higher level of evidence. Finally, the trial is open label so subjects and researchers can know what procedure was undertaken to treat.

The final review paper will be submitted to a peer-reviewed journal for publication and presented at relevant conferences (Supplementary Table 1).

## Data Availability

The raw data supporting the conclusions of this article will be made available by the authors, without undue reservation.
