# Supplementary figures and images for: Efficacy and Safety of Camrelizumab in Combination with Docetaxel + S-1 Sequenced by Camrelizumab + S-1 for Stage III (PD-1+/MSI-H/EBV+/dMMR) Gastric Cancer: Study Protocol for a Single-Center, Prospective, Open-Label, Single-Arm Trial
Source: Front Surg. 2022 Jun 28;9:917352. doi: 10.3389/fsurg.2022.917352 (PMC9274117; doi:10.3389/fsurg.2022.917352)

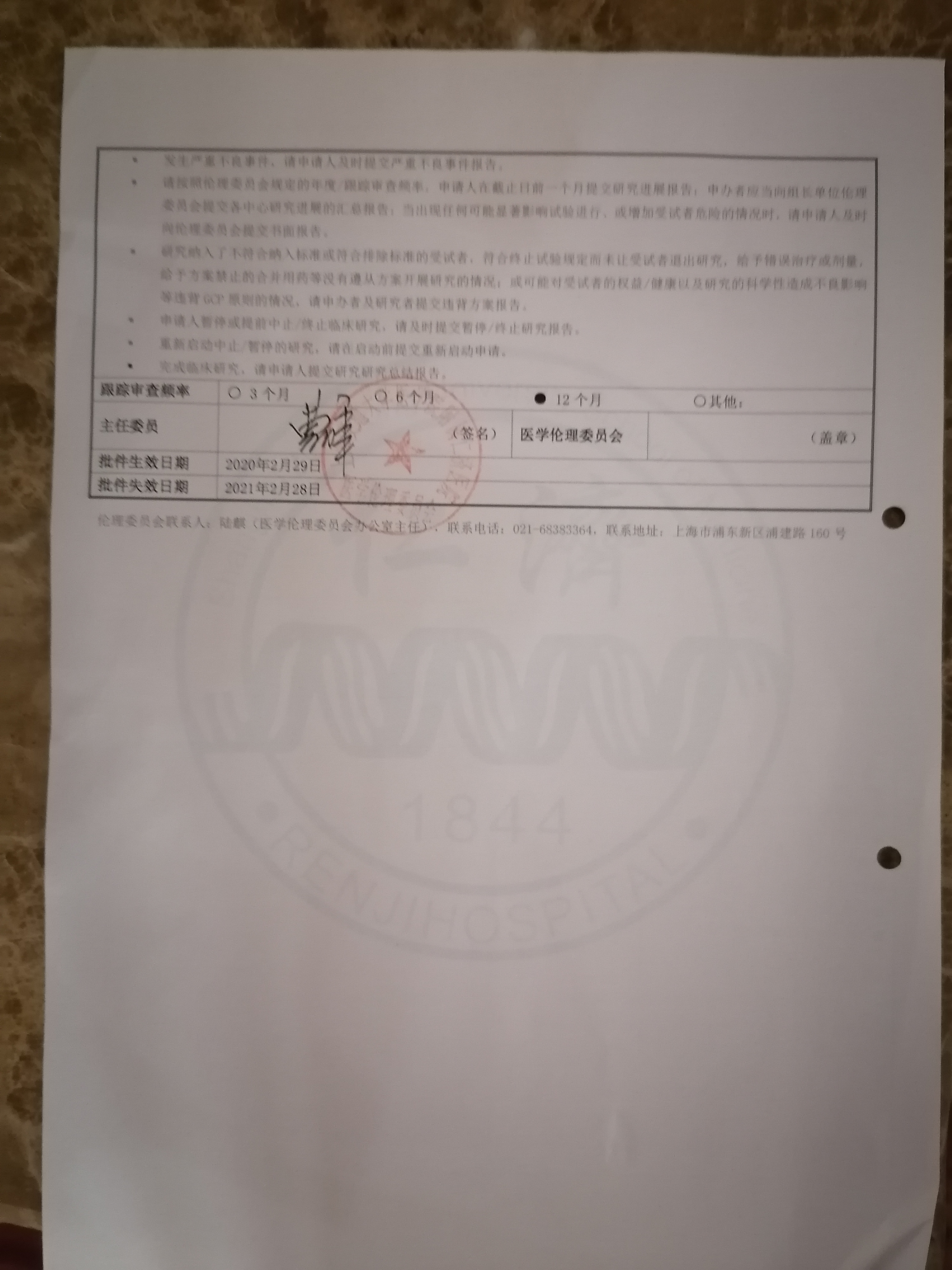

Supplement: Supplementary file 1 [file Image_1_v1.jpeg]

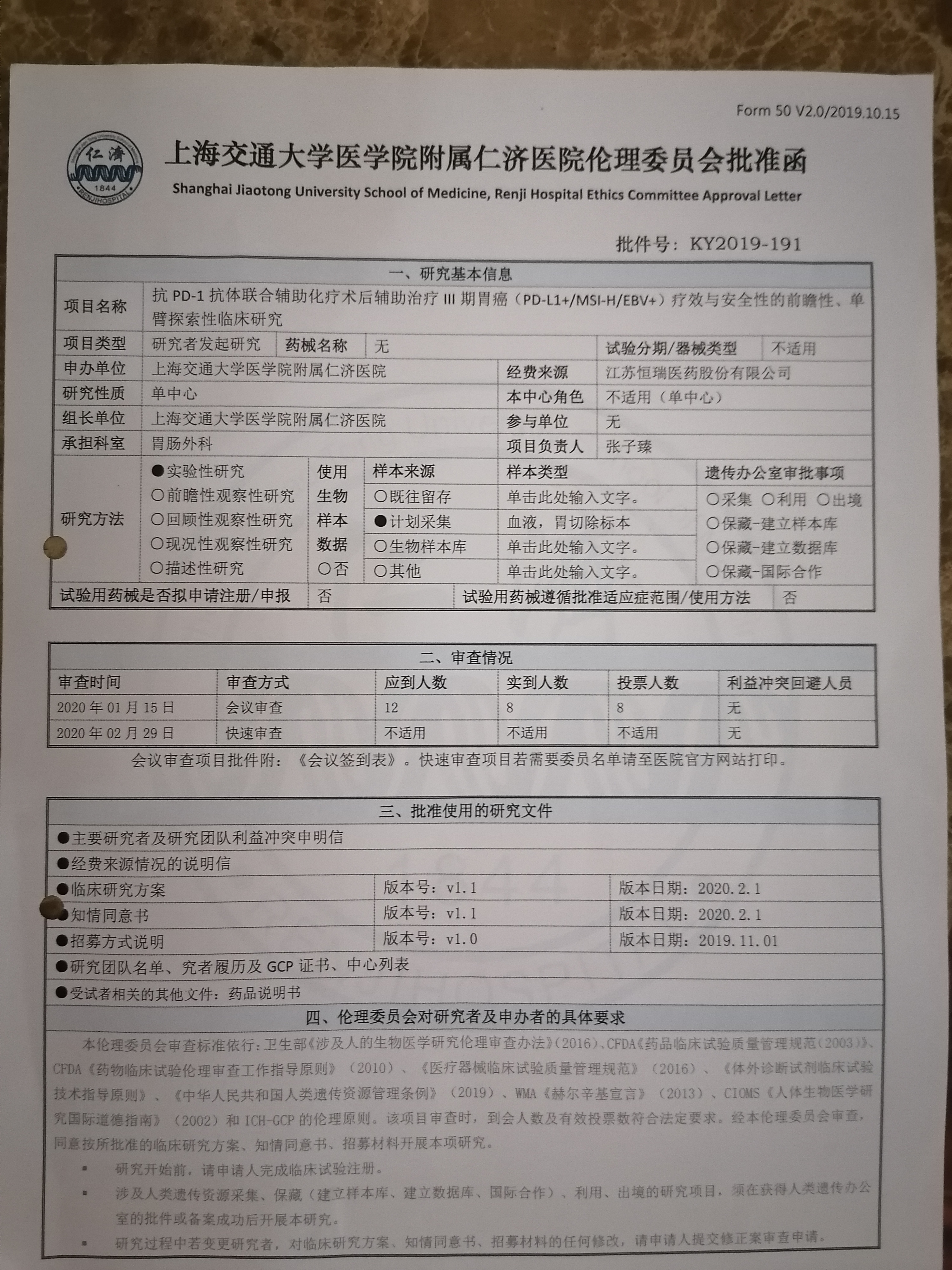

Supplement: Supplementary file 2 [file Image_2_v1.jpeg]
