# Supplementary material for: Efficacy and Safety of Camrelizumab in Combination with Docetaxel + S-1 Sequenced by Camrelizumab + S-1 for Stage III (PD-1+/MSI-H/EBV+/dMMR) Gastric Cancer: Study Protocol for a Single-Center, Prospective, Open-Label, Single-Arm Trial
Source: Front Surg. 2022 Jun 28;9:917352. doi: 10.3389/fsurg.2022.917352 (PMC9274117; doi:10.3389/fsurg.2022.917352)
Supplement: Supplementary file 4 [file Table_3_v1.docx]

Shanghai Jiaotong University School of Medicine,Renji Hospital Ethics CommitteeApproval Letter

Petition number:KY2019-191

1.Basic information about study

Project name: Prospective, single-arm exploratory clinical study to explore the efficacy and safety of anti-PD-1 antibody combined with adjuvant chemotherapy in patients with stage III(PD-L1+/MSI-H/EBV +)gastric cancer patient

Project type: Researcher-initiated study Medical instrument: N/A Trial staging/Instrument Type: N/A

Bidding agency: Renji Hospital, School of Medicine, Shanghai Jiao Tong University Source of funding: Jiangsu Hengrui

Study character: Single center The central role: N/A

Unit leader: Renji Hospital, School of Medicine, Shanghai Jiao Tong University Participating institutions: No

Responsible department: Department of Gastrointestinal Surgery Project leader: Zizhen Zhang

Research methodology: Experimental Study Biological sample data: Plan to collect Blood, Gastrectomy specimen

Whether an experimental drug is intended to be registered/declared: NO Experimental drug following the approved range of indications/use: No

2.Status of review

Time of review Mode of review People supposed to come Actual number Voter numbers Avoid conflicts of interest

2020-01-15 Conference review 12 8 8 No

2020-02-29 Quick review N/A N/A N/A No

3.Research papers approved for use

- Declaration of conflict of interest by the principal investigator and the research team
- Information letter on sources of funding
- Clinical research program V1.1
- Informed consent V1.1
- Description of Recruitment Methods
- List of research teams, Researcher’s resume, GCP certificate, Central list
- Other documents related to the subject: Drug instructions

4.Specific requirements of the Ethics Committee for researchers and sponsors

The criteria for this ethics committee’s review are based on: Ministry of Health“Ethical Review of biomedical research involving human beings“(2016), CFDA “Code for quality control of drug clinical trial”(2003), CFDA “Guiding Principles for ethical review of drug clinical trials”(2010), “Code for quality management of clinical trials of medical devices”(2016), “Technical Guidelines for clinical trials of in vitro diagnostic reagents”, “Regulations of the People’s Republic of China on the administration of human genetic resources”(2019), WMA “Declaration of Helsinki”(2013), CIOMS “International Ethical Guidelines for human biomedical research”(2002),ICH-GCP Ethical principles. When the project is examined, the number of participants and the number of valid votes met the statutory requirements. Upon review by this ethics committee, agree to conduct this project in accordance with the approved clinical research program, informed consent, and recruitment materials.

- Applicants are requested to complete the clinical trial registration prior to the start of the study.
- Research projects involving the collection, preservation (establishment of sample bank, establishment of database, international cooperation) , utilization, and exit of human genetic resources shall be carried out after obtaining the approval or filing of the office of Human Genetics.
- In case of changes in the course of the study, the applicant is requested to submit an amendment review application for any changes to the clinical research program, informed consent form, or recruitment materials.
- If a serious adverse event occurs, the applicant is requested to submit a serious adverse event report in a timely manner.
- According to the frequency of the annual follow-up report stipulated by the Ethics Committee, the applicant shall submit a research progress report one month before the deadline; the applicant shall submit a summary report of the research progress of each center to the Ethics Committee of the Group Leader Unit; The applicant is requested to submit a written report to the ethics committee in a timely manner in the event of any circumstances that may significantly affect the conduct of the experiment or increase the risk to the subject.
- The study included subjects who did not meet the criteria or met the exclusion criteria, met the criteria for termination of the study but did not withdraw the subjects from the study, and were given the wrong treatment or dose, non-compliance with programme studies, such as the administration of drugs prohibited by the programme, Or a violation of the GCP principles such as a possible adverse impact on the rights/health of the subject and the science of the study. When this happens, the applicant is requested to submit a proposal report to the researcher.
- If the applicant suspends or terminates the clinical study in advance. Please submit the study report in time.
- To restart discontinued clinical studies, applicants are requested to submit research reports in time.
- Upon completion of the clinical study, the applicant is requested to submit a summary report of the study.

Frequency of tracking: 12 months
