# Supplementary material for: Efficacy and Safety of Camrelizumab in Combination with Docetaxel + S-1 Sequenced by Camrelizumab + S-1 for Stage III (PD-1+/MSI-H/EBV+/dMMR) Gastric Cancer: Study Protocol for a Single-Center, Prospective, Open-Label, Single-Arm Trial
Source: Front Surg. 2022 Jun 28;9:917352. doi: 10.3389/fsurg.2022.917352 (PMC9274117; doi:10.3389/fsurg.2022.917352)
Supplement: Supplementary file 5 [file Table_4_v1.docx]

The partial results of this study

| ID | Age | BMI | ECOG | Sex | Enrollment time | Immunohistochemical | Number of chemo |
| --- | --- | --- | --- | --- | --- | --- | --- |
| Patient 1 | 59 | 21.45 | 1 | Male | June 2,2020 | PD-L1 20% | 8 |
| Patient 2 | 68 | 29.03 | 1 | Male | June 6,2020 | PD-L1 10% | 15 |
| Patient 3 | 65 | 19.15 | 1 | Male | June 23,2020 | DMMR PD-L1 95% | 16 |
| Patient 4 | 43 | 19.72 | 1 | Female | July 2,2020 | PD-L1 20% | 17 |
| Patient 5 | 71 | 19.33 | 1 | Male | July 7,2020 | PD-L1 5% | 16 |
| Patient 6 | 53 | 19.03 | 1 | Female | July 21,2020 | PD-L1 20% | 4 |
| Patient 7 | 37 | 21.88 | 1 | Female | Sept 26,2020 | PD-L1 30% | 11 |
| Patient 8 | 34 | 19.83 | 1 | Female | Nov 7,2020 | PD-L1 20% | 17 |
| Patient 9 | 68 | 23.14 | 1 | Female | Dec 8,2020 | PD-L1 5% | 15 |
| Patient 10 | 48 | 30.49 | 1 | Male | Dec 21,2020 | DMMR | 18 |
| Patient 11 | 64 | 24.97 | 1 | Female | Jan 11,2020 | PD-L1 5% | 15 |
| Patient 12 | 58 | 23.05 | 1 | Female | April 7,2021 | PD-L1 15% | 9 |
| Patient 13 | 65 | 20.57 | 1 | Male | April 9,2021 | PD-L1 40% | 17 |
| Patient 14 | 75 | 24.54 | 1 | Male | June 17,2021 | PD-L1 20% | 12 |
